# Stomatal Features, Specific Leaf Area and Water Relations in Three Pistachio Cultivars

**DOI:** 10.3390/plants15030494

**Published:** 2026-02-05

**Authors:** Sara Álvarez, Lidia Núñez, José Manuel Mirás-Avalos

**Affiliations:** 1 Instituto Tecnológico Agrario de Castilla y León (ITACyL), Ctra. Burgos, 119, 47071 Valladolid, Spain; nunramli@itacyl.es; 2 Misión Biológica de Galicia (MBG-CSIC), Sede Santiago, Avda. de Vigo s/n, Campus Vida, 15705 Santiago de Compostela, Spain

**Keywords:** gas exchange, *Pistacia vera* (L.), stomatal control

## Abstract

Stomatal traits are determinants of plant water relations and may differ among cultivars within a given species. These features have been rarely studied in tree crops, such as pistachio (*Pistacia vera* L.), the cultivation of which is expanding in several Mediterranean countries due to its economics revenues. Therefore, this study aims at characterizing several stomata features (length, width, surface, and density) in three pistachio cultivars (Golden Hills, Lost Hills, and Kerman) growing in Central Spain. In addition, the relationships between these traits and gas exchange and stem water potential (Ψ_s_) measurements were explored over the 2025 growing season. Kerman trees had more negative Ψ_s_ values than the other cultivars when atmospheric demand was high, which translated into lower stomatal conductance and net photosynthesis values. This coincided with lower stomatal density and specific leaf area in Kerman. However, stomata were bigger in Kerman than in the other cultivars. When compared over the course of the day, stomatal conductance in the abaxial leaf surface was, approximately, 70% greater than that observed on the adaxial side of the leaves in the three cultivars. These findings provide relevant insights for understanding the regulation of water relations in this species, which may serve for planning new plantations.

## 1. Introduction

Pistachio (*Pistacia vera* L.) trees are native to Western Asia, but they are widely grown in the Mediterranean, with large increases in planting surface in the South of Europe. For instance, the total planted area in Spain was negligible in 1990, but it is currently 78,000 ha [1]. This increase in pistachio acreage has been caused by uncertainties in the future economic support of the European Union to traditional crops, such as olive and grapevine, and the use of low-fertility soils [2]. In certain Spanish regions where no restrictions (winter chill or water availability) are expected, such as Castilla y León (central Spain), the surface devoted to pistachio is expanding steadily [3]. As of 2022, 2835 ha are devoted to pistachio in this region, where it is gaining relevance due to its high economic revenues, helping to fix populations in rural areas [4]. However, this expansion in acreage comes with an increase in the use of irrigation, which must be precisely applied to maximize yield and avoid the overexploitation of water resources [5]. In addition, the selected plant material is the main driver for the success and viability of these plantations [6,7], and it may have different stomatal control, hence responding differently to a given water management method [8], making irrigation scheduling difficult.

Rootstocks exert a significant impact on pistachio response to water stress and rehydration, and some rootstocks induce less stomatal control than others [2,9]. In this sense, the UCB-1 rootstock, a cross between *Pistacia atlantica* and *Pistacia integerrima*, is preferred under irrigation conditions [10]. In contrast, few reports focused on the potential differences among pistachio scions [8,11], as most research has been carried out on the Kerman cultivar [2,9,12] because of its economic relevance. Nevertheless, pistachio cultivars differ in physiological traits including heat and chill requirements and the duration of the growing cycle [13]. For instance, ‘Kerman’ is the most widely grown pistachio cultivar in Spain, but it has high requirements of heat, while ‘Golden Hills’ and ‘Lost Hills’ have a shorter cycle due to their lower cold and heat requirements [14,15]. These traits, along with the fact that these two cultivars are reported to be more productive and with a higher percentage of split nuts than ‘Kerman’ [14,15], make them adequate for cultivation in regions prone to spring frosts and with short summers, such as Castilla y León, in Spain [4].

A recent study [11] assessed the water relations in three pistachio cultivars (‘Kerman’, ‘Golden Hills’, and ‘Lost Hills’) in an orchard subjected to a moderate water stress, revealing that, under the same watering regime, ‘Kerman’ reached the lowest values of several physiological indicators: midday stem water potential (Ψ_s_), leaf osmotic potential at full turgor (Ψ_os_), stomatal conductance (g_s_) and net photosynthesis rate (P_n_), while exhibiting the highest intrinsic water use efficiency (WUE_i_). Moreover, Núñez et al. [11] showed that the relationships between Ψ_s_ and g_s_, despite being significant in all cases, differed among cultivars, suggesting that ‘Kerman’ has a stricter stomatal control than the other two cultivars. However, anatomical traits such as density of stomata and specific leaf area were not measured and could, at least partly, explain the observed differences in the response of these three pistachio cultivars.

Indeed, stomata play a crucial role in regulating gas exchange between plants and their environment, as their number, size and aperture define the gas exchange potential of a given plant species [16]. The regulation of these stomata is affected by several environmental factors [17]. Drought is one of the most detrimental abiotic factors, as it decreases leaf water potential, limits stomatal aperture, and suppresses photosynthesis-related gene expression [18]. The earliest and most critical response to drought is stomatal closure, which is controlled by a complex signaling network [19]. However, stomatal closure to a given level of drought may vary in intensity among species and within a given species. Partial stomatal closure reduces transpirational water loss but, simultaneously, limits CO_2_ uptake, thereby restricting photosynthetic capacity [20]. Moreover, stomatal density varies among species and even within varieties, thereby enabling plants to adapt to specific environments or climatic conditions [21]. Studies in pistachio revealed significant differences in stomatal density in Iranian cultivars [22], but most of these works were performed in laboratory conditions and using rootstocks, and field studies are lacking. Moreover, previous studies have focused on the isolated characterization either of stomatal physiology or stomatal anatomy, but combining these approaches offer advantages for both trait development and modeling [23]. The stomatal number and size, along with other anatomical features of the stomatal cells, are fixed after leaf expansion [24] and are relevant traits for determining total pore area (stomatal area index, SAI) and maximum stomatal conductance (g_smax_) [25]. Additionally, stomatal opening and closure regulates instantaneous CO_2_ uptake and water loss relative to changes in the environmental conditions (temperature, light, humidity) [26,27,28]. To date, research has mainly focused on the stomata within the lower leaf surface (abaxial) to understand the mechanisms and regulation of stomatal opening and closure [16]. However, several species are amphistomatous and produce stomata also on the adaxial (upper) leaf surface, with pistachio being among these species [29,30]. Despite this, few studies have focused on the different features of adaxial and abaxial stomata in tree crops [31]. Therefore, the aim of the current study was to assess the relevance of stomatal traits (including a differentiation between abaxial and adaxial leaf surfaces) in the response, in terms of water relations, to deficit irrigation of three pistachio cultivars (‘Kerman’, ‘Golden Hills’ and ‘Lost Hills’) grown in an orchard located in Central Spain and managed under the same conditions.

## 2. Results

### 2.1. Weather Conditions and Soil Water Dynamics During the Study Period

**Figure 1:**
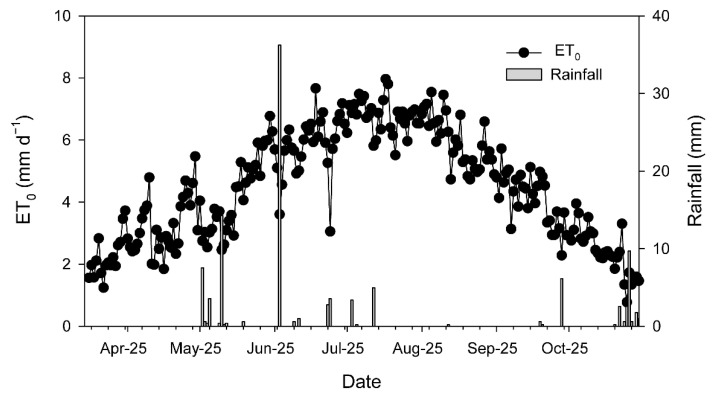
Evolution of daily reference evapotranspiration (ETo) and rainfall recorded at the agrometeorological station of Torrecilla de la Orden (https://www.Inforiego.org/opencms/opencms (accessed on 10 December 2025)) during the 2025 growing season (from March to October).

Over the 2025 growing season (from 1 March to 31 October), mean temperature was 16.8 °C, ranging from −2.9 °C to 40.5 °C. Total rainfall was 189.1 mm, while effective rainfall amounted to 77.7 mm. The reference evapotranspiration (ETo) was 1053.7 mm and translated into 958 mm crop evapotranspiration (ETc). The season was dry (only 34 days with rainfall > 1 mm), and most of the rainfall events occurred between April and June, with an event of around 36 mm in the beginning of June (Figure 1, Table A1). No rainfall was registered from the end of July to the end of September (Table A1), whereas daily ETo over that period ranged between 5 and 8 mm (Figure 1). The amount of water applied through irrigation was 3100 m^3^ ha^−1^, which, adding the effective rainfall over the season, corresponded to 41% of the ETc.

**Figure 2:**
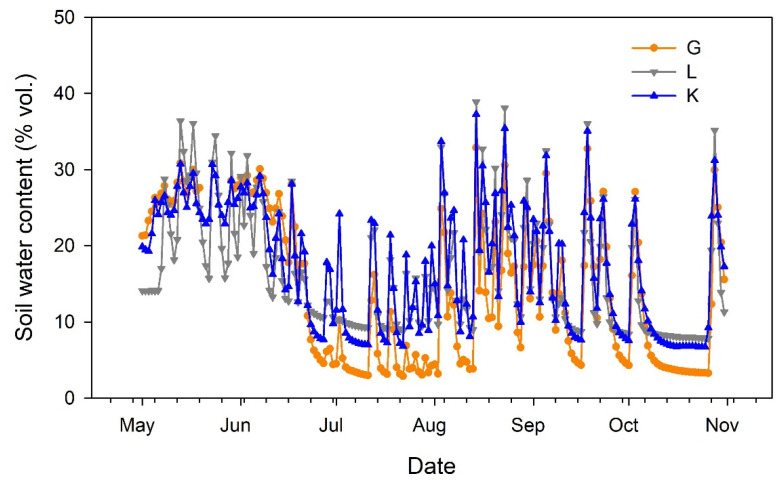
Evolution of soil water content at 40 cm depth in three pistachio cultivars during the experimental period (May to October 2025). G = Golden Hills; L = Lost Hills; K = Kerman.

Soil water content at 40 cm depth (Figure 2) reflected the irrigation and rainfall events, showing similar dynamics for the three pistachio cultivars. The sensor located in the portion of the orchard devoted to the Lost Hills cultivar provided lower values in May. Then, lower soil water content was recorded for the Golden Hills cultivar during July. Afterwards, soil water content tended to balance among cultivars (Figure 2).

### 2.2. Water Relations in Three Different Pistachio Cultivars: Midday Stem Water Potential and Gas Exchange Parameters

**Figure 3:**
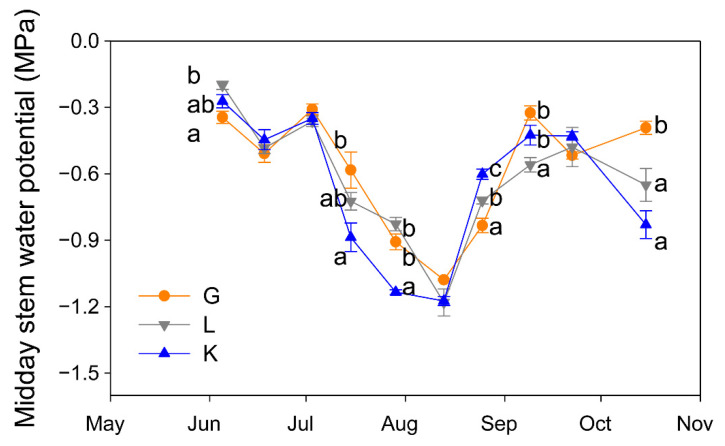
Evolution of midday stem water potential in three pistachio cultivars during the experimental period (May to October 2025). Different letters indicate significant differences among cultivars on a given date, according to Duncan’s test (*p* < 0.05). When no letters appear on a given date, significant differences among cultivars were not detected. G = Golden Hills; L = Lost Hills; K = Kerman.

The three pistachio cultivars experienced a similar seasonal pattern in their water status (Figure 3). However, Ψ_s_ values differed among cultivars in most of the measurement dates. At the beginning of the season, Golden Hills showed the most negative Ψ_s_ value, but this was early in the season and Ψ_s_ was greater than −0.4 MPa in all cultivars, indicating no signs of water stress. In contrast, when the atmospheric demand was higher (in July), significantly lower Ψ_s_ values were observed in the Kerman cultivar, although by mid-August and September this trend was reversed and Kerman showed less negative Ψ_s_ values than Golden Hills and Lost Hills (Figure 3). After harvest, Golden Hills showed the least negative Ψ_s_ values (Figure 3).

**Figure 4:**
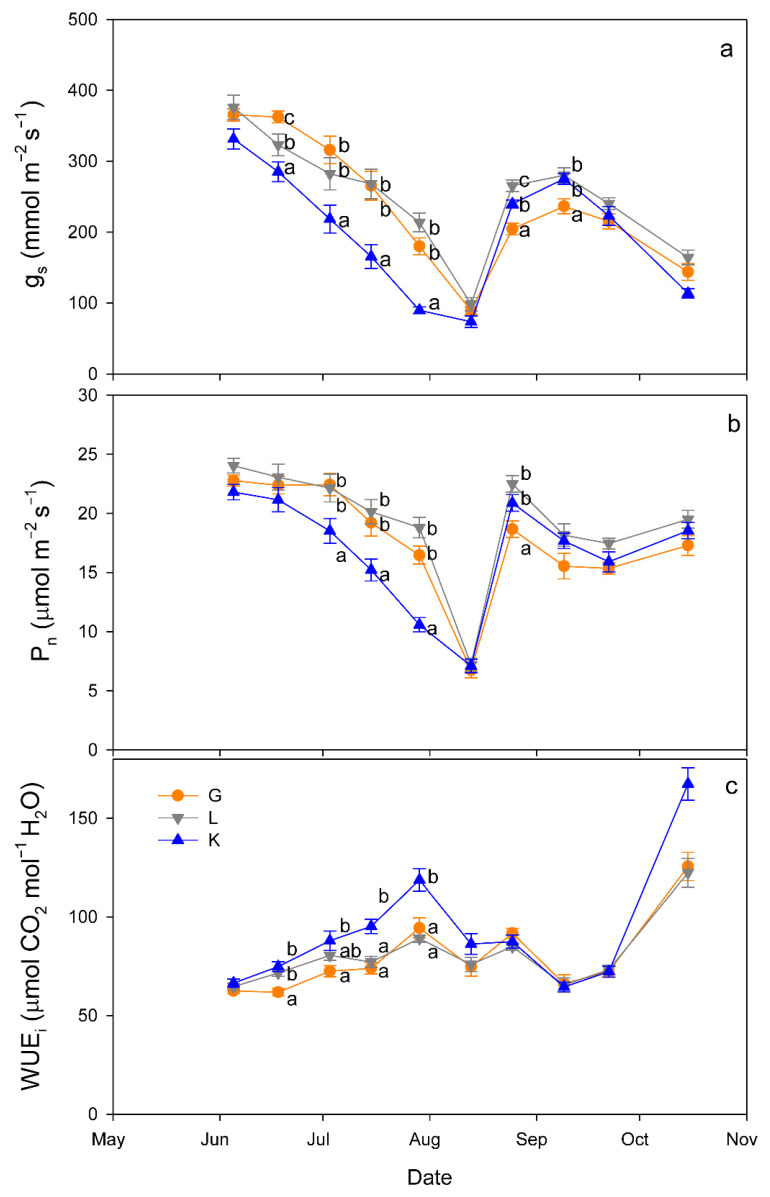
Evolution of stomatal conductance (g_s_; (**a**)), net photosynthesis rate (P_n_; (**b**)) and intrinsic water use efficiency (WUE_i_; (**c**)) in three pistachio cultivars during the experimental period (June to October 2025). Different letters indicate significant differences among cultivars on a given date, according to Duncan’s test (*p* < 0.05). When no letters appear on a given date, significant differences among cultivars were not detected. G = Golden Hills; L = Lost Hills; K = Kerman.

**Figure 5:**
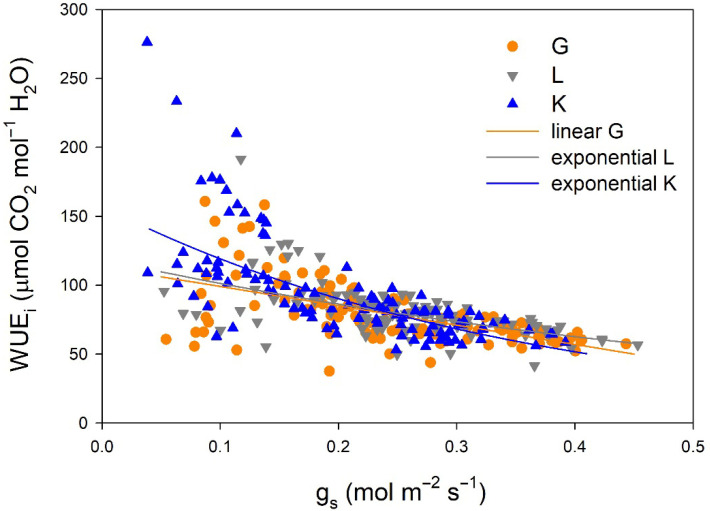
Relationships between stomatal conductance (g_s_) and intrinsic water use efficiency (WUE_i_) in three pistachio cultivars during 2025. G = Golden Hills; L = Lost Hills; K = Kerman. The depicted equations are the following: WUE_i_ = 113.11 − 140.15 × g_s_ (R^2^ = 0.334; *p*-value < 0.001) for Golden Hills, WUE_i_ = 118.86 × e^(−1.599×gs)^ (R^2^ = 0.342; *p*-value < 0.001) for Lost Hills, and WUE_i_ = 157.36 × e^(−2.782×gs)^ (R^2^ = 0.497; *p*-value < 0.001) for Kerman.

Regarding gas exchange parameters, stomatal conductance (g_s_) values were significantly lower in the Kerman cultivar than in the Golden Hills and Lost Hills cultivars from mid-May to the end of July, but in mid-August and early September, the lowest values were observed in Golden Hills (Figure 4a). A similar pattern was observed for net photosynthesis (P_n_) rate (Figure 4b). In summary, g_s_ values ranged from 38 to 392 mmol m^−2^ s^−1^ for Kerman, while they varied between 52 and 453 mmol m^−2^ s^−1^ for Golden Hills and Lost Hills. The combination of lower g_s_ and P_n_ meant that Kerman trees had the highest intrinsic water use efficiency (WUE_i_) values until the middle of the growing season (Figure 4c). The highest WUE_i_ values were observed when g_s_ was between 50 and 150 mmol m^−2^ s^−1^, decreasing as the degree of stomatal aperture increased (Figure 5).

The relationships between g_s_ and WUE_i_ differed among cultivars (Figure 5). A linear equation was fitted to the data from Golden Hills, while exponential equations were adjusted to data from Lost Hills and Kerman. Despite a slight difference at high g_s_ values, the equations fitted to data from Golden Hills and Lost Hills were similar. In contrast, Kerman showed higher WUE_i_ at lower g_s_ when compared to the other two cultivars (Figure 5).

Specific leaf area was significantly greater in Golden Hills (7.18 mm^2^ mg^−1^) than in the other two cultivars (6.24 and 6.02 mm^2^ mg^−1^ for Lost Hills and Kerman, respectively).

### 2.3. Stomatal Traits and Stomatal Conductance in the Abaxial and Adaxial Side of the Leaves

**Figure 6:**
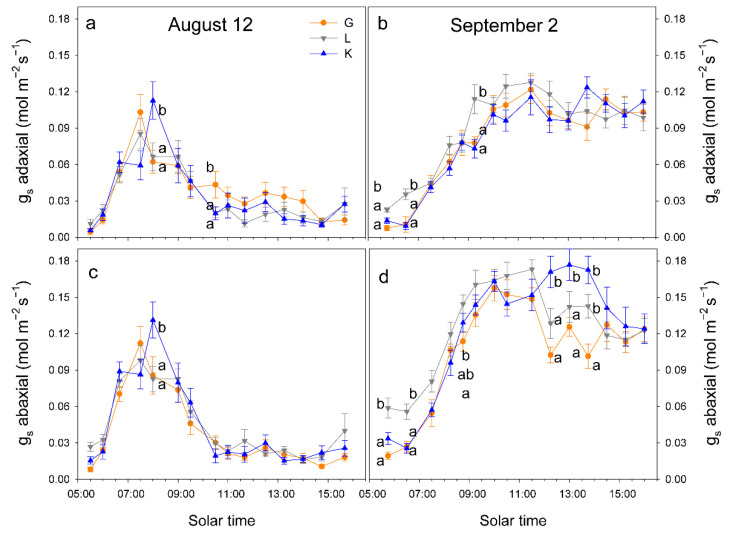
Daily pattern of stomatal conductance (g_s_) in the abaxial and adaxial side of the leaves from three pistachio cultivars on 12 August 2025 (**a**,**c**) and on 2 September 2025 (**b**,**d**). Different letters indicate significant differences among cultivars at a given time according to Duncan’s test (*p* < 0.05). When no letters appear on a given date, significant differences among cultivars were not detected. G = Golden Hills; L = Lost Hills; K = Kerman.

**Figure 7:**
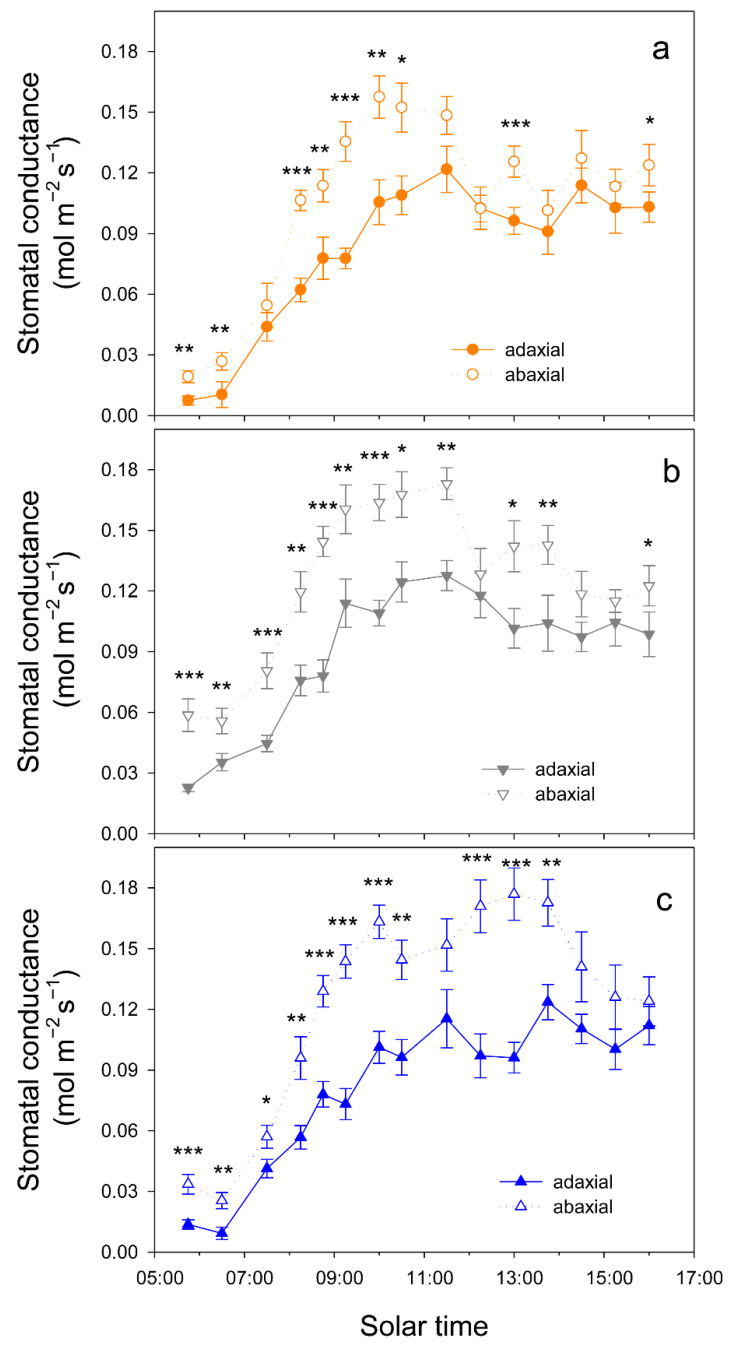
Daily pattern of stomatal conductance in the adaxial and abaxial side of the leaves from three pistachio cultivars, i.e., Golden Hills (**a**), Lost Hills (**b**) and Kerman (**c**), on 2 September 2025. Asterisks indicate significant differences between leaf sides at a given time according to Student’s test: * *p* < 0.05; ** *p* < 0.01; *** *p* < 0.001.

When atmospheric demand, measured as vapor pressure deficit (VPD), was high (on 12 August, VPD = 7 kPa at solar noon), trees tended to close stomata before midday and significant differences in g_s_ among cultivars were only detected at 10 h, when g_s_ was higher in Kerman on both sides of the leaves (Figure 6). In contrast, when atmospheric demand was lower (on 2 September, VPD = 3 kPa at solar noon), g_s_ values were higher in Lost Hills than in the other two cultivars until 12 h, while in the afternoon, g_s_ was higher in Kerman trees (Figure 6). There were significant differences between leaf sides in terms of g_s_ in all cultivars, with greater values on the abaxial side than those measured on the adaxial side (Figure 7).

Stomatal density differed among cultivars, with Kerman having the lowest and Golden Hills the highest stomatal density (Table 1). This was caused by significant differences among cultivars in both the stomatal density in the abaxial and adaxial sides of the leaf (Table 1). All cultivars had more stomata per mm^2^ in the abaxial than in the adaxial side of their leaves (Table 1). In addition, stomatal density followed the following rank order: Kerman < Lost Hills < Golden Hills.

Significant differences in stomatal length, width and density were detected among pistachio cultivars (Table 2). Stomata were longer and wider in Kerman leaves, and smaller in Golden Hills (Table 2). Moreover, stomata were significantly longer in the abaxial than in the adaxial side of the leaves of the three pistachio cultivars (Table 2). However, only Kerman showed significant differences in stomatal width and density between leaf sides (Table 2).

### 2.4. Osmotic Potential and Color Features

**Figure 8:**
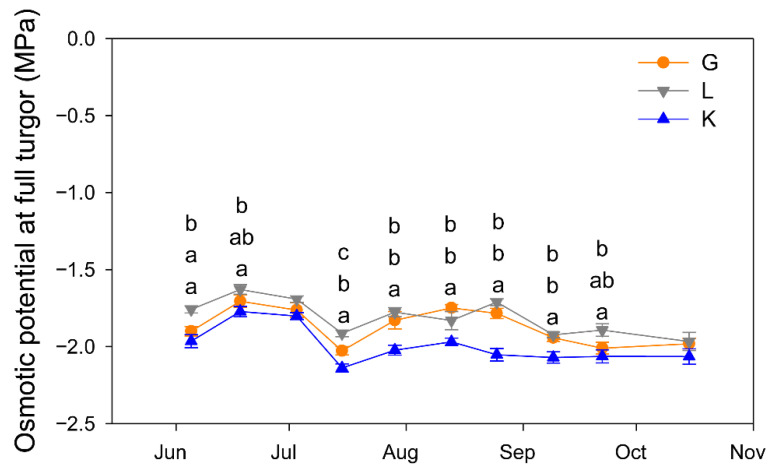
Seasonal pattern of leaf osmotic potential at full turgor in three pistachio cultivars. Different letters indicate significant differences among cultivars on a given date according to Duncan’s test (*p* < 0.05). When no letters appear on a given date, significant differences among cultivars were not detected. G = Golden Hills; L = Lost Hills; K = Kerman.

The three cultivars showed a similar seasonal pattern of Ψ_os_, but Kerman tended to produce the most negative values, being significantly lower than those of Golden Hills and Lost Hills, which only differed on a couple of dates over the growing season (Figure 8).

Regarding color features (Table 3), leaves from the Lost Hills cultivar showed a higher lightness (L*) value in both sides of the leaf, whereas hue (h°) values were greater in Kerman leaves (Table 3). In terms of chroma (C*), significant differences were only observed in the adaxial side of the leaves, with Lost Hills having the greatest values. Moreover, differences in color parameters were observed between leaf sides (Table 3). The lower L*, C* and h° values were recorded in the adaxial side of the leaves of the three pistachio cultivars, confirming the darker and less vivid green color of the upper side (adaxial) compared with the lower side (abaxial) of the leaves.

## 3. Discussion

The current study pointed out differences in the stomatal features of three pistachio cultivars that may modulate their specific responses to water stress. For instance, Kerman showed bigger but less dense stomata than Golden Hills and Lost Hills. These features translated into an earlier closure of stomata over the growing season in Kerman when compared to the other cultivars. Moreover, this coincided with more negative Ψ_s_ values in Kerman. However, when the atmospheric demand declined (by the end of August), g_s_ and Ψ_s_ values in Kerman recovered to similar levels as those measured in the other cultivars. These results confirm previous findings regarding the differences in water relations among pistachio cultivars [11].

The stomatal response of pistachio trees varies between two different strategies [9]: (i) substantial and prolonged stomatal opening during the day, and (ii) partial stomatal closure at mid-morning. In the current study, both strategies were on display, as the stomatal aperture of the three cultivars reduced considerably from around 9 h solar time when the atmospheric demand was high (12 August), and g_s_ remained below 0.05 mol m^−2^ s^−1^ during the day. Conversely, when the atmospheric demand was low (2 September), stomata were substantially open, and g_s_ remained at about 110 mol m^−2^ s^−1^ during the day. This suggests the efficient transpiration control in this species, avoiding unnecessary water losses [12]. Moreover, clear differences were detected among cultivars when g_s_ was high, and, usually, Kerman showed greater values than Golden Hills and Lost Hills in the afternoon, while the opposite occurred early in the morning, when the atmospheric demand was low. This seems to contradict previous observations in which a stricter control of stomata was reported for Kerman trees [11], but it can be explained by the lower atmospheric demand observed in the current study that may have masked some differences among cultivars.

Rapid stomatal responses to environmental change play a key role in maintaining water movement from soil to plant [32]. A major actor in this control is stomatal size, which is negatively correlated with the sensitivity to increasing drought [33]. In fact, larger stomas close more slowly and have been proven to potentially cause hydraulic dysfunctions under drought [33]. Despite the fact that only a mild water stress occurred during the current study, Kerman response to increasing drought conditions fitted this pattern, as it had the largest stomas from the three pistachio cultivars studied and had the lowest Ψ_s_ values when environmental conditions over the growing season were drier, since it tended to close stomata later in the day than the other two cultivars. Conversely, small stomata can open and close more rapidly, and their general association with high densities allow for rapid increases in g_s_, maximizing CO_2_ diffusion into the leaf during favorable conditions for photosynthesis [34]. In this sense, the Golden Hills cultivar had the smallest stomas (22% and 14% smaller than those from Kerman and Lost Hills, respectively) and tended to close earlier in the day when compared to those of Kerman. Previous studies on pistachio have shown different responses to drought depending on the cultivar [35]. Sensitivity to water stress in pistachio cultivars reflects in stomatal size, among other traits, suggesting that reducing stomatal size is a protective adaptation to minimize water loss in this species. In our work, the combination of greater stomata and more negative Ψ_s_ values in Kerman suggests that this cultivar maintains its photosynthetic capacity under mild water stress, while Golden Hills and Lost Hills tended to be more protective and minimize water loss.

The three cultivars had stomata on both sides of the leaves, as previously reported for the *P. vera* species [29]. However, the stomatal density observed in the current study was greater than that previously reported for this species [29], likely due to the high intra-cultivar variability of stomatal traits [21]. In addition, the ratio of the number of stomas in the adaxial and abaxial leaf surfaces ranged from 0.66 (in Kerman) to 0.77 (in Lost Hills), slightly lower than the value of 0.8 previously reported for *P. vera* [29]. However, as expected, the number of stomas found on the adaxial leaf surface of the pistachio cultivars studied was substantially higher than that of other species of the *Pistacia* genus, such as *P. atlantica* [30], *P. integerrima* and *P. terebinthus* [29]. These differences may be explained by the wide range of environmental conditions in which these studies were carried out and that significantly affect stomatal traits [34].

Regarding leaf color, the abaxial sides of the pistachio leaves were lighter and brighter, as indicated by the higher L*, C* and hue values, due to lower light exposure and a different pigment distribution [36]. The adaxial/abaxial distinction reflects structural and functional differences, which affect overall leaf color (hue, lightness and saturation). The adaxial side of a leaf, the light-facing side, is often darker green due to higher chlorophyll concentrations in palisade cells for light capture in photosynthesis, while the abaxial side, the shaded one, receiving less direct light, is often lighter (+L*), matte (+C*), grayish-green (+h°) and with more stomata for gas exchange, featuring spongy mesophyll [16,37]. Color differences were detected among cultivars in our study, with lower L* and C* values and higher h° values recorded in the leaves of Kerman, which is the cultivar with more scattered stomata. In this sense, high positive correlations between leaf color and leaf chlorophyll content have been reported for several species [38].

In the three pistachio cultivars considered in this work, their g_s_ values were significantly greater in the abaxial than in the adaxial surface of the leaves. However, despite these differences, the contribution of the adaxial stomata to the total g_s_ was high, especially early in the morning and late in the afternoon, when it was greater than 45% of the total g_s_, while at mid-morning, it reached 35–38% depending on the cultivar. Further studies are required to determine the operation mechanisms of each surface, as they seem to be independent in some species [31,39]. The adaxial stomatal conductance may support greater evaporative leaf cooling to maintain optimal leaf temperatures for photosynthesis, as reported in other species [39].

The Ψ_os_ values measured in the current study varied between −1.7 and −2.1 MPa, being similar to those reported for the Kerman cultivar grafted onto *P. terebinthus* in Central–South Spain [40]. As previously reported [11], Kerman trees had a higher degree of osmotic adjustment since they showed lower Ψ_os_ values over the growing season than Golden Hills and Lost Hills trees. This active accumulation of solutes in the plant as a response to increasing drought conditions is a mechanism of resistance to dehydration [41] that varies among species and cultivars [42]. In water-limited environments, cultivars with a high degree of osmotic adjustment tended to produce greater yields [43], although this trend has not been consistently observed [44].

It must be noted that, during the study period, pistachio trees experienced only mild water stress conditions as, despite the reduction in Ψ_s_ and g_s_ values during the summer months (especially in July and August), the −1.5 MPa threshold that is reported to cause yield penalties was never surpassed [9].

Finally, the approach for determining stomatal traits used in the current work has some limitations. For instance, the unavoidable presence of air bubbles that interferes with image clarity, as well as causing the irregularity of stomatal imprints, which can make data analysis tedious, and thus increase the time required to measure stomatal traits [45]. In addition, analyzing images manually is subject to human error and can result in inaccurate data. Another major limitation in using the nail polish method is the time it takes to acquire data [45]. This factor prevents the application of the method on a large scale to screen stomatal traits in large populations [45]. Previous studies have mentioned these drawbacks, pointing out the difficulty in capturing stomatal width reliably and thus opting for other methods to estimate this trait [46]. However, in the current study, we compared stomatal traits among three pistachio cultivars at a specific point in time and we assumed that the force of impression or leaf detachment does not affect stomatal pore width. This was ensured by the fact that the same person carried out the nail polish imprints on the leaves. Indeed, this is also subject to uncertainties, but for the comparative purpose of the current study, it can be assumed. Future research could make use of new low-cost microscopes to monitor stomatal pore opening in real time under field conditions [47].

## 4. Materials and Methods

### 4.1. Description of the Study Site

The experimental site is a commercial pistachio orchard located in Carpio, Valladolid, Spain (41°12′ N, 5°5′ W, 759 m elevation). Pistachio trees of three different cultivars (Golden Hills, Lost Hills and Kerman) were planted in 2012 at 5 m × 6 m spacings (333 trees ha^−1^). Moreover, two masculine/pollinizing cultivars were spread over the plot (11% of the total number of trees): ‘Peter’ and ‘Randy’. Trees were grafted onto the University of California at Berkeley I (UCB1) rootstock. Trees were irrigated using two lateral pipes per tree row, each located 0.7 m from the tree on either side of the planting line, with seven 2.2 L h^−1^ pressure-compensating emitters per tree. The orchard was drip-irrigated from April to October using a computer-controlled system and following the criteria of the farmer. All cultivars received the same amount of water and management practices over the study period (2025) and during the previous two years (2023 and 2024). In 2025, the amount of water applied was 3447 m^3^ ha^−1^, which added to the effective precipitation, corresponded to 41% of the estimated ETc.

Soil texture at the experimental site is sandy loam (66% sand, 25% clay), and its pH is alkaline (8.7), while electrical conductivity (0.26 dS m^−1^) and organic matter content (0.45%) are low. The climate of the region is continental Mediterranean, with an annual mean temperature of 12.2 °C and rainfall of 352.5 mm per year. Precipitation is mostly distributed outside a two-month (July and August) summer drought period.

The experiment consists of three replicates of four trees per cultivar laid out in a completely randomized design (12 trees per treatment). These trees were similar in size, as the trunk diameters at the end of the season were 15.16 ± 0.19 cm for Golden Hills, 15.37 ± 0.15 cm for Lost Hills and 15.16 ± 0.12 cm for Kerman.

### 4.2. Measurements

Daily records of weather variables (including maximum and minimum temperature, relative humidity, rainfall and ETo, among others) were collected from the nearest agrometeorological station (VA02—Torrecilla de la Orden, at 9 km from the experimental orchard) managed by the InfoRiego service of the Castilla y León Government (https://www.inforiego.org/opencms/opencms (accessed on 10 December 2025)). Additionally, from March to October (the pistachio growing season), a sensor (MX2301, HOBO, Onset Computer Corporation, Bourne, MA, USA) was deployed in the experimental plot to monitor ambient temperature and relative humidity.

Soil water content (in m^3^ m^−3^) was monitored with SMT-100 sensors (TRUEBNER GmbH, Neustadt an der Weinstraße, Germany) at a 40 cm depth (one sensor per cultivar). The general calibration for mineral soils proposed by the manufacturer was used to transform the electrical signal provided by the sensors into measures of soil water content.

From May to October, tree water status was monitored periodically by measuring Ψ_s_ and gas exchange parameters in 12 trees per cultivar. A pressure chamber (Mod. 3000, Soil Moisture Equipment Co., Santa Barbara, CA, USA) was used to measure Ψ_s_ biweekly in leaves that were previously bagged with aluminum foil 2 h prior to the measurements. Leaves were collected from inside the canopy, close to the trunk or the main scaffolds. Since pistachio trees exude turpentine, making the measurement of Ψ_s_ difficult, a piece of blotting paper was placed above the petiole cut-off to facilitate measurements as turpentine does not moisten paper, but xylem water does. For assessing g_s_ and P_n_, on the same trees as those used for Ψ_s_ measurements, a portable gas analyzer (LI-COR 6800, LI-COR Inc., Lincoln, NE, USA) was used. Leaf gas exchange parameters were measured every two weeks on 12 attached, young, fully expanded leaves per cultivar, placed in a 2 cm^2^ leaf cuvette at mid-morning (around 10:00 GMT). The P_n_/g_s_ ratio was used as an estimation of the WUE_i_.

In addition, to obtain a diurnal pattern, on 12 August and 2 September 2025, g_s_ was measured every 40–50 min over the course of the day (from 5:00 to 16:00 h solar time), using a handheld porometer/fluorometer (LI-600, LI-COR Inc., Lincoln, NE, USA) in 12 trees per cultivar. In this case, the measurement was first performed on the adaxial side of the leaf, and then the leaf was inverted, and the same procedure was conducted on the abaxial leaf surface. Leaf total stomatal conductance was obtained by adding the conductance recordings of the abaxial and adaxial surfaces.

Leaf osmotic potential at full turgor (Ψ_os_) was measured every two weeks in seven trees per cultivar, using excised leaves with their petioles placed in distilled water overnight to reach full saturation. Leaves from the Ψ_os_ measurements were then frozen in liquid nitrogen (−196 °C) and stored at −30 °C for one week. After thawing, Ψ_os_ was measured in the extracted (mechanical pressing) sap using a Wescor 5520 vapor pressure osmometer (Wescor, Logan, UT, USA), as described by Gucci et al. [48].

Stomatal density was determined on nail polish imprints [49] taken from the adaxial and abaxial leaf surfaces, at the widest part of the leaf blade (middle region) and avoiding major veins. For doing this, 48 leaves from 12 trees per cultivar were used, and three fields of view were measured randomly in each surface, following the distinction between the adaxial and abaxial epidermis. Stomata were counted under a light microscope (Leica DM5000B, Leica Microsystems, Wetzlar, Germany) at a magnification of 400×, and stomatal density was expressed as the number of stomas per mm^2^. For each of the images, the size of three representative stomata (width and length) was measured in micrometers (μm), and the stomatal surface area was calculated using the formula of an ellipse (stomatal width × stomatal length × π/4).

Leaf area was assessed in 60 leaves per cultivar, by employing a leaf area meter (Delta-T Devices Ltd., Cambridge, UK). Subsequently, leaves were subjected to oven-drying at 80 °C until a constant weight was achieved, enabling the measurement of their respective dry weights (DWs). Specific leaf area (SLA) was determined in these leaves and calculated by dividing the leaf area by the corresponding leaf dry weight. Finally, color coordinates, namely lightness (L*), chroma (C*) and hue angle (h°) [50], were measured in the abaxial (lower) and adaxial (upper) leaf surfaces with a Minolta CR-10 colorimeter (Konika Minolta, Tokyo, Japan), using 40 leaves per cultivar.

### 4.3. Statistical Analysis

Normality and homoscedasticity of the data were checked using the Shapiro–Wilk and Bartlett tests, respectively. Analysis of variance followed by Duncan’s test were used for determining the effect of pistachio cultivar on the measured parameters. Differences between the sides of the leaf were separated with Student’s test. Relationships between g_s_ and WUE_i_ for each cultivar were assessed using linear and nonlinear regression. Statistical analyses were conducted with the R software version 4.3.3 [51].

## 5. Conclusions

The current work highlighted some differences in the stomatal traits of three pistachio cultivars growing under the same conditions in the field, which translated into distinct responses to increasing drought. Kerman, the pistachio cultivar most widely grown in Spain, had less dense (up to 12.5% difference) and bigger (up to 28% difference) stomata than Golden Hills and Lost Hills. This feature partly explains the stricter stomatal control previously reported for Kerman. In the current work, stomatal conductance values ranged from 38 to 392 mmol m^−2^ s^−1^ for Kerman, while they varied between 52 and 453 mmol m^−2^ s^−1^ for Golden Hills and Lost Hills. Net photosynthesis rate was approximately 30% lower in Kerman than in the other cultivars during July and August. Additionally, the contribution of the adaxial stomata to the total g_s_ of the leaves was high, reaching up to 48% in the morning and late in the afternoon in the three cultivars. These findings suggest that the Kerman cultivar responds more rapidly to water deficit than the other two cultivars, leading to an earlier stomatal closure and, consequently, a lower assimilation of CO_2_, which can lead to yield penalties. Therefore, irrigation management should be tailored to each specific pistachio cultivar. Further research is required to elucidate the operation mechanisms of stomatal functioning in each surface of the pistachio leaves. Specifically, further studies should focus on the role of adaxial stomatal function and its comparison with abaxial stomatal behavior to better understand the physiology of different pistachio cultivars. Additionally, further studies focusing specifically on adaxial stomata may help to identify genes that control stomatal distribution between leaf surfaces in pistachio.

## Figures and Tables

**Table 1:** Stomatal density (mean ± standard error) in the abaxial and adaxial sides of the leaf and on average per leaf in three pistachio cultivars. Different lower-case letters in the columns indicate significant differences between cultivars according to Duncan’s test (*p* < 0.05). Different upper-case letters in the row indicate significant differences in the sides of the leaf for a given cultivar according to Duncan’s test (*p* < 0.05).

Cultivar	Stomatal Density (Stomata mm^−2^)		
	Leaf	Adaxial	Abaxial
Golden Hills	242.3 ± 4.5 c	205.9 ± 3.2 bA	279.4 ± 3.3 bB
Lost Hills	229.1 ± 4.1 b	200.1 ± 3.1 bA	258.2 ± 3.4 aB
Kerman	215.4 ± 4.9 a	171.1 ± 2.4 aA	259.4 ± 3.8 aB

**Table 2:** Stomatal dimensions (mean ± standard error) in the abaxial and adaxial sides of the leaf and on average per leaf in three pistachio cultivars. Different lower-case letters indicate significant differences among cultivars according to Duncan’s test (*p* < 0.05), and different upper-case letters indicate differences between leaf sides for a given cultivar according to Student’s test (*p* < 0.05).

Parameter	Side	Golden Hills	Lost Hills	Kerman
Stomatal length (mm)	Leaf	23.5 ± 0.2 a	24.8 ± 0.2 b	26.2 ± 0.1 c
	Adaxial	22.8 ± 0.2 aA	23.2 ± 0.2 bA	26.0 ± 0.2 cA
	Abaxial	24.2 ± 0.2 aB	25.8 ± 0.2 bB	26.5 ± 0.2 cB
Stomatal width (mm)	Leaf	13.4 ± 0.1 a	14.9 ± 0.1 b	15.6 ± 0.1 c
	Adaxial	13.5 ± 0.2 a	14.8 ± 0.2 b	16.0 ± 0.2 bB
	Abaxial	13.4 ± 0.1 a	14.9 ± 0.1 b	15.1 ± 0.1 cA
Stomatal surface (mm^2^)	Leaf	255.0 ± 3.1 a	297.6 ± 3.5 b	326.5 ± 3.6 c
	Adaxial	249.6 ± 4.7 a	288.2 ± 5.4 b	336.6 ± 5.7 cB
	Abaxial	260.4 ± 4.1 a	307.0 ± 4.6 b	319.2 ± 4.2 cA

**Table 3:** Color features (mean ± standard error) in the adaxial and abaxial sides of the leaves from three pistachio cultivars. Different lower-case letters indicate significant differences among cultivars according to Duncan’s test (*p* < 0.05), and different upper-case letters indicate differences between leaf sides for a given cultivar according to Student’s test (*p* < 0.05).

Leaf Side	Parameter	Golden Hills	Lost Hills	Kerman
Adaxial	L*	38.13 ± 0.21 abA	38.46 ± 0.23 bA	37.45 ± 0.31 aA
	C*	15.22 ± 0.25 aA	15.37 ± 0.31 aA	14.45 ± 0.41 aA
	h°	93.27 ± 0.03 aA	93.23 ± 0.04 aA	99.18 ± 0.12 bA
Abaxial	L*	39.72 ± 0.23 bB	41.31 ± 0.31 cB	38.04 ± 0.30 aB
	C*	18.01 ± 0.26 bB	19.70 ± 0.30 cB	15.70 ± 0.39 aB
	h°	93.46 ± 0.04 aB	93.47 ± 0.05 aB	99.51 ± 0.10 bB

L*: lightness; C*: chroma; h°: hue angle.

## Data Availability

The raw data supporting the conclusions of this article will be made available by the authors on request.

## Abbreviations

The following abbreviations are used in this manuscript:

Ψ_s_	Stem water potential
g_s_	Stomatal conductance
Ψ_os_	Leaf osmotic potential at full turgor
P_n_	Net photosynthesis rate
WUE_i_	Intrinsic water use efficiency
g_smax_	Maximum stomatal conductance
SAI	Stomatal area index
ETo	Reference evapotranspiration
ETc	Crop evapotranspiration
VPD	Vapor pressure deficit
L*	Lightness
C*	Chroma
h°	Hue angle
UCB	University of California at Berkeley
DW	Dry weight
SLA	Specific leaf area

## Appendix A

The rainfall events that occurred over the pistachio growing season (March to October) recorded in the weather station nearest to the experimental orchard are displayed in Table A1.

**Table A1:** Daily rainfall amounts recorded at the agrometeorological station of Torrecilla de la Orden during the pistachio growing season (from March to October).

Date	Rainfall (mm)	Effective Rainfall (mm)
1 March 2025	6.34	2.9
2 March 2025	1.98	0
6 March 2025	1.19	0
7 March 2025	1.19	0
8 March 2025	2.57	0
9 March 2025	3.17	0.32
10 March 2025	2.57	0
11 March 2025	11.68	6.8
16 March 2025	1.98	0
18 March 2025	5.54	2.29
20 March 2025	1.98	0
21 March 2025	3.76	0.83
22 March 2025	8.51	4.54
24 March 2025	6.53	3.06
4 April 2025	2.38	0
11 April 2025	6.14	2.75
12 April 2025	2.97	0.14
16 April 2025	1.78	0
18 April 2025	5.74	2.45
19 April 2025	2.57	0
30 April 2025	9.5	5.28
2 May 2025	7.52	3.82
5 May 2025	3.56	0.66
10 May 2025	14.26	8.58
3 June 2025	36.23	22.09
23 June 2025	2.77	0
24 June 2025	3.56	0.66
3 July 2025	3.37	0.49
12 July 2025	4.95	1.83
28 September 2025	6.14	2.76
21 October 2025	2.57	0
24 October 2025	2.57	0
25 October 2025	9.7	5.4
28 October 2025	1.78	0
